# Multimode Magneto-Optical Fiber Based on Borogermanate Glass Containing Tb^3+^ for Sensing Applications

**DOI:** 10.3390/ma18204736

**Published:** 2025-10-16

**Authors:** Douglas F. Franco, Steeve Morency, Younès Messaddeq, Marcelo Nalin

**Affiliations:** 1 São Paulo State University–UNESP–Institute of Chemistry, Araraquara 14800-060, SP, Brazil; younes.messaddeq@copl.ulaval.ca (Y.M.); marcelo.nalin@unesp.br (M.N.); 2 Centre d’optique, photonique et laser (COPL), Université Laval (UL), 2375 Rue de la Terrasse, Québec, QC G1V 0A6, Canada

**Keywords:** optical fiber, glasses, magneto-optical materials

## Abstract

A multimode magneto-optical fiber based on Tb^3+^-containing borogermanate glass was designed, fabricated, and characterized, aiming at potential sensing applications. There are continuing challenges in the development of single-mode (SMF) or multimode (MMF) optical fibers doped with rare-earth (RE) ions and exhibiting high Verdet constants, related to devitrification of the precursor glass. Most RE-doped glass compositions are not suitable as precursors for core-cladding fiber production due to devitrification processes and consequent poor optical quality. Application as Faraday rotators is limited by the intrinsically low Verdet constant of silica (~0.589 rad T^−1^ m^−1^ at 1550 nm and 0.876 rad T^−1^ m^−1^ at 1310 nm). Borogermanate glasses are good candidates for manufacturing optical fibers due to their excellent potential to solubilize high concentrations of Tb^3+^ ions as well as satisfactory thermal stability. In this work, a magneto-optical core-cladding borogermanate fiber with a 227 μm diameter was fabricated, with characterization using differential scanning calorimetry (DSC), thermomechanical analysis (TMA), viscosity measurements, M-lines spectroscopy, UV-Vis-NIR absorption spectroscopy, the cut-back technique, and magneto-optical measurements. The measured numerical aperture (NA) was 0.183, with minimum attenuation of 13 dB m^−1^ at 1270 nm. The Verdet constant (V_B_) reached −6.74 rad T^−1^ m^−1^ at 1330 nm.

## 1. Introduction

Magneto-optical (MO) materials have attracted significant attention due to their versatility in optical and MO applications, including optical isolators [1,2], transducers [2,3], and sensors [2,4,5]. In recent years, various crystalline garnets [6] and vitreous materials, such as glasses [7,8,9,10], glass ceramics [11], and optical fibers [12,13,14], have been developed for magneto-optical applications.

Among these materials, garnets have emerged as particularly promising due to their very high Verdet constant (V_B_) values in the visible and near-infrared (NIR) spectral regions [15]. Terbium gallium garnet (TGG) [16] and yttrium iron garnet (YIG) [17] single crystals are the MO materials that have received most attention for practical applications, with reported V_B_ values of −134 rad T^−1^ m^−1^ at 632 nm (TGG) and 304 rad T^−1^ m^−1^ at 1550 nm (YIG) [15,17]. These garnets exhibit enhanced Faraday rotation angles, resulting in high Verdet constants, and are typically synthesized by the Czochralski crystal growth method [18]. However, their MO properties are limited because Faraday rotation is constrained by the physical crystal length (optical path), and the crystals are typically only available with dimensions on millimeter or centimeter scales. To address this drawback, MO fibers represent an attractive alternative, since their extended optical path lengths can provide enhanced MO sensitivity.

Significant challenges remain in the exploration of new glass compositions containing high concentrations of paramagnetic elements, such as rare-earth (RE) ions, with high V_B_ values in the visible and NIR regions. Furthermore, not all glass compositions are suitable for the fabrication of optical fibers, often lacking sufficient thermal stability, adequate mechanical properties, and the optical quality required for practical applications as waveguides for light transmission. In recent years, borogermanate glasses based on heavy metal oxides (HMOs) have attracted attention due to their ability to solubilize high concentrations of paramagnetic RE ions without crystal or cluster formation. This property enables the development of interesting magneto-optical materials [7,9,10,13,19]. Technologically, the optical fiber manufacturing process requires glass precursors with high thermal stability parameters (ΔT > 150 °C), avoiding devitrification, for the fiber drawing process. One of the advantages of the borogermanate glass matrix is its high ΔT values, which are typically greater than 200 °C. However, borogermanate glasses are characterized by inherent water absorption and high viscosity. This can lead to striae and structural imperfections in glass samples, causing optical signal losses greater than those observed in commercial silica fibers [7]. Few studies have reported the design and fabrication of glass preforms for core-cladding MO fibers containing high concentrations of RE ions and presenting high V_B_ values [11,20,21,22]. Heavy metal oxide (HMO) glasses containing high concentrations of RE ions typically tend to crystallize during the fiber drawing process, preventing successful optical fiber formation. Nevertheless, the literature reports the development of optical fibers doped with Eu^3+^, Ho^3+^, Pr^3+^, and Tb^3+^ [11,20,21,22,23].

Cruz et al. described the MO characterization of a standard silica optical fiber in the wavelength range 458–1523 nm [23]. The silica fiber exhibited V_B_ values of 3.25 and 0.54 rad T^−1^ m^−1^ at 633 and 1523 nm, respectively. Huang et al. used europium-doped silica to obtain a core-cladding fiber by a modified chemical vapor deposition (MCVD) method [12], where Eu^3+^ ions were incorporated in the fiber core by thermal vaporization, resulting in core and cladding diameters of 9 and 122 μm, respectively. The calculated V_B_ value for the fiber was −4.563 rad T^−1^ m^−1^ at 660 nm, representing a two-fold increase, compared to the value of 2.413 rad T^−1^ m^−1^ for a single-mode fiber (SMF). Liu et al. reported the fabrication and characterization of a holmium-doped silica fiber obtained by the MCVD method [20]. The measured V_B_ values of the core-cladding fiber were −3.915 and −1.287 rad T^−1^ m^−1^ at 1310 and 1550 nm, respectively, representing enhancements of 4.6 and 1.6 times compared to the standard SMF. Linganna and coauthors fabricated a Pr^3+^-doped glass fiber with a 125 μm diameter using glass with the composition SiO_2_-l_2_O_3_-B_2_O_3_--Pr_6_O_11_ for all-optical isolator applications. The Pr^3+^-doped fiber exhibited a V_B_ of −17.28 rad T^−1^ m^−1^ at 650 nm [21].

Among the paramagnetic RE ions, Tb^3+^ has been widely employed to increase the V_B_ values of glasses and optical fibers. This approach is particularly valuable because, as noted previously, standard SMFs exhibit a significantly lower V_B_ in telecom wavelength regions (especially at 1550 nm) [12]. The terbium ion, with electronic configuration of 4f^8^ → 4f^7^5d ^8,9^, has among the highest magnetic moments (μ_eff_ = 9.5–9.72 μB) and magnetic susceptibilities (J = 6, g = 1.46) of all the f-block ions [19].

Sun et al. reported the MO characterization of a single-mode phosphate fiber containing 25 wt% Tb^3+^ doping in the core and 6 wt% La^3+^ doping in the cladding [24]. The fabricated fiber presented core and cladding diameters of 4.5 and 120 μm, respectively, with a propagation loss of 0.12 dB cm^−1^ at 980 nm. The measured V_B_ reached −6.2 rad T^−1^ m^−1^ at 1053 nm, representing an approximately six-fold enhancement compared to a standard silica fiber. Subsequently, Sun et al. developed a compact all-fiber Faraday isolator, utilizing 65 wt% Tb^3+^-doped silicate fiber, achieving a V_B_ of −32 rad T^−1^ m^−1^ at 1053 nm, corresponding to a twenty-seven-fold enhancement compared to a silica fiber [25]. Franco et al. recently reported the fabrication of two different Tb^3+^-containing borogermanate no-core fibers [7]. The first study concerned the development of a no-core MO fiber based on the GeO_2_-B_2_O_3_-Al_2_O_3_-Na_2_O-PbO-Tb_4_O_7_ (GBANPb-xTb) glass system, with a composition containing 4 mol% Tb_4_O_7_ (equivalent to 21.4 wt% Tb doping). The GBANPb-4Tb fiber exhibited a V_B_ of −5.89 rad T^−1^ m^−1^ at 1550 nm, representing a ten-fold enhancement compared to a commercial SMF. Franco et al. also fabricated a second no-core fiber using a GeO_2_-B_2_O_3_-Al_2_O_3_-10NaO-BaO-Tb_4_O_7_ (BGB-xTb) glass composition with 8 mol% Tb_4_O_7_ (35 wt% Tb^3+^) [22]. The BGB-8Tb fiber exhibited a V_B_ of −22.8 rad T^−1^ m^−1^ at 1050 nm and −9.5 rad T^−1^ m^−1^ at 1550 nm in the NIR range, representing nineteen-fold and sixteen-fold enhancements, respectively, compared to a commercial single-mode fiber.

This work presents the core-cladding glass preform design; manufacturing; and thermal, optical, and magnetic characterization of a magneto-optical core-cladding fiber based on borogermanate glass containing a high concentration of terbium oxide. The glass preform and optical fiber were characterized using differential scanning calorimetry (DSC), thermomechanical analysis (TMA), viscosity measurements, M-lines spectroscopy, UV-Vis-NIR absorption spectroscopy, the cut-back fiber loss method, and magneto-optical measurements.

## 2. Materials and Methods

### 2.1. Bulk Glass Preparation

The two bulk glass precursors for the core and cladding, based on Tb^3+^-doped borogermanate, were prepared according to the conventional melt-quenching method. The precursor chemicals used were germanium oxide (99.9%, Sigma-Aldrich, Burlington, MA, USA), boron oxide (99.9%, Sigma-Aldrich), aluminum oxide (99.9%, Sigma-Aldrich), sodium carbonate (99.9%, Sigma-Aldrich), barium carbonate (99.9%, Sigma-Aldrich), and terbium (III/IV) oxide (99.9%, Sigma-Aldrich). The precursors were stoichiometrically weighed out to give 10 g masses of materials with molar composition 92 (41GeO_2_-25B_2_O_3_-4Al_2_O_3_-10Na_2_O-20BaO)-8Tb_4_O_7_ (BGB-8Tb) (cladding composition) and 91 (41GeO_2_-25B_2_O_3_-4Al_2_O_3_-10Na_2_O-20BaO)-9Tb_4_O_7_ (BGB-9Tb) (core composition), according to the procedure reported by Franco et al. [22]. The glass components were homogenized in an agate mortar, loaded into a platinum crucible, and melted at 1400 °C for 2 h in a resistive furnace under atmospheric conditions. After homogenization, the melts were cooled in preheated stainless-steel molds at 50 °C below the glass transition temperature for 5 h to minimize mechanical stress. After cooling, BGB-8Tb and BGB-9Tb glasses with thicknesses of approximately 3 mm were obtained. The glasses were polished using silicon carbide (SiC) paper prior to optical and magneto-optical characterizations.

### 2.2. Core-Cladding Glass Preform Preparation

**Figure 1:**
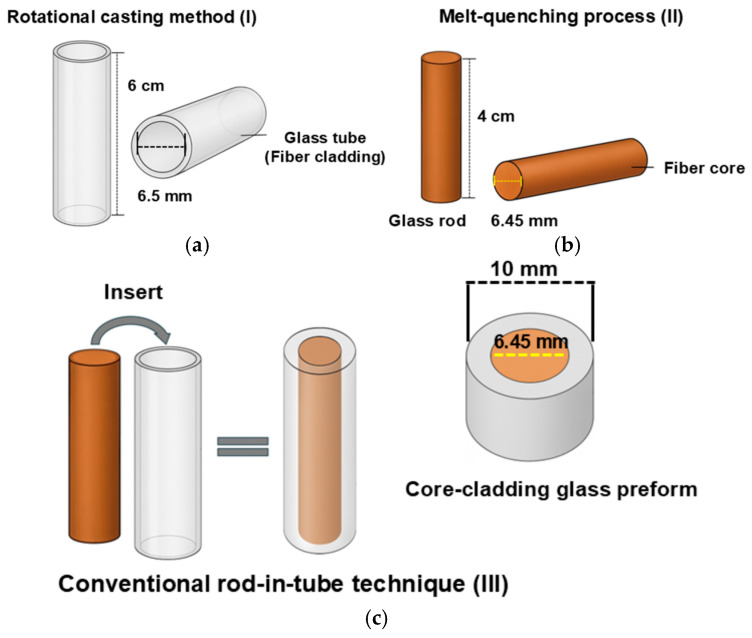
Fabrication steps: (**a**) rotational casting method for cladding preparation (step I); (**b**) prepared cladding tube and core rod (step II); and (**c**) conventional rod-in-tube assembly and final core-cladding glass preform (step III).

The selection of BGB-9Tb and BGB-8Tb glass compositions for the core and cladding, respectively, was based on an analysis of thermal stability parameters (ΔT) and refractive index values at 532 nm (Table 1 in Section 3). The experimental procedure for fabricating the core-cladding glass preform and drawing the optical fiber was divided into three steps, as described below:

 **(I)**  **Cladding preparation:** Firstly, the cladding fiber was prepared using the glass composition containing 8 mol% Tb_4_O_7_ (BGB-8Tb). A glass tube measuring 6 cm in length, with an outer diameter of 10 mm and inner diameter of 6.5 mm, was fabricated using the rotational casting method (Figure 1a). For this, 25 g of BGB-8Tb glass was melted at 1400 °C for 1 h in a platinum crucible. After melting, the cladding composition was cast into a preheated cylindrical stainless-steel mold (maintained at 50 °C below T_g_) mounted in a rotational casting furnace. Rotation was immediately performed for 15 s at 2500 rpm, forming the glass tube by centrifugal action (Figure 1a). The rotation system was then switched off, and thermal annealing of the glass tube was performed for 6 h at 550 °C (below T_g_) to minimize structural stress of the tube. The glass tube was then cooled at a slow cooling rate of 0.5 °C min^−1^ until reaching room temperature. In the final step, the external surface of the glass tube was polished with silicon carbide (SiC) papers (600, 800, 1200, 2400, and 4000 mesh) using an automatic polishing machine, followed by a final polishing with an aluminum oxide suspension.

 **(II)**  **Core preparation:** The core fiber was fabricated from a glass rod preform (4 cm length × 6.45 mm diameter) containing 9 mol% of Tb_4_O_7_ (BGB-9Tb), according to the experimental parameters detailed in Section 2.1 (Figure 1b). For this, 20 g of the BGB-9Tb glass composition was melted for 1 h in a platinum crucible at 1400 °C in a resistive furnace. The melt was cast into a cylindrical preheated mold maintained at 50 °C below T_g_ (564 °C), followed by thermal annealing for 6 h. The glass rod was cooled to room temperature at a rate of 0.5 °C min^−1^. A rod measuring 6.45 mm in diameter and 4 cm in length was prepared. The glass rod was polished using the same procedure described above for the glass tube.
 **(III)**  **Conventional rod-in-tube technique:** The third step in the preparation of the core-cladding glass preform involved the insertion of the BGB-9Tb glass rod (core) into the BGB-8Tb glass tube (cladding) to obtain the final assembled core-cladding glass preform system (Figure 1c). The glass rod was fitted into the glass tube, ensuring complete surface contact, since improper fitting of the core within the glass tube could lead to morphological defects in the resulting optical fiber.

For drawing into a fiber, the core-cladding glass preform was mounted in the furnace at the top of a fiber drawing tower. The fiber drawing process started at around 730–750 °C, and the length of the manufactured core-cladding fiber was around 10 m. The optical fiber was coated with a low-index UV-cured poly(methyl methacrylate) (PMMA) polymer to enhance the mechanical properties and provide protection.

**Table 1:** Characteristic temperatures of the BGB-8Tb and BGB-9Tb samples (glass transition, T_g_; crystallization onset, T_x_; dilatometric glass transition, T_gd_; dilatometric softening, T_s_), fiber drawing temperatures (T_fib_), thermal stability parameters (ΔT = T_x_ − T_g_), and thermal expansion coefficients (α).

Sample	T_g_ (±2 °C)	T_x_ (±2 °C)	ΔT (±4 °C)	T_gd_ (±2 °C)	T_s_ (±2 °C)	α_(25–500 °C)_ (±0.05) (10^−6^ K^−1^)	T_fib_
BGB-8Tb	599	913	314	583	628	10.3	734
BGB-9Tb	614	896	282	590	650	10.2	744

### 2.3. Characterization Techniques

Differential scanning calorimetry (DSC) measurements of the BGB-8Tb and BGB-9Tb glasses were performed using a Netzsch DSC Pegasus 404 F3 instrument (Selb, Germany). Approximately 15 mg portions of the glasses were loaded into platinum crucibles and heated from 20 to 1000 °C at a rate of 10 °C min^−1^ under a nitrogen atmosphere (30 mL min^−1^ flow rate). The measurement errors were ±2 °C (T_g_ and T_x_) and ±4 °C (ΔT).

Thermal mechanical analysis (TMA) measurements were performed using a Netzsch TMA F1 Hyperion instrument (Selb, Germany), with cylindrical samples (10 mm diameter × ∼10 mm height) of the BGB-xTb glasses (x = 8 and 9 mol% Tb_4_O_7_) heated at a rate of 5 °C min^−1^ under a mechanical load of 0.02 N. The dilatometric glass transition temperature (T_gd_), softening point (T_s_), and linear thermal expansion coefficient (α) were determined from the TMA curves.

Viscosity measurements of the BGB-xTb glasses were performed using a Theta US parallel plate viscometer (San Jose, CA, USA) in the range from 10^10^ to 10^7^.^5^ poise. Cylindrical samples (10 mm diameter × ∼10 mm height) were positioned between two parallel silica disks in the furnace and heated to 400 °C at a rate of 5 °C min^−1^ under a compressive load of 300 mg. From 400 °C to 700–780 °C, the heating rate was increased to 10 °C min^−1^. The log viscosity was calculated using the DilaSoft Version 3 software. Linear behaviors of the viscosity-temperature curves were observed within this viscosity range, providing an acceptable approximation, as reported previously by Skopak et al. [26] and Galstyan et al. [27]. The fiber drawing temperature was estimated by linear extrapolation of the viscosity curve for 10^7^ poise, with an associated error of ±5 °C. The experimental error of the viscosity measurements was approximately ±0.2 log(poise).

Morphological and cross-sectional analyses of the optical fiber were performed by scanning electron microscopy (SEM) imaging using a Quanta 3D FEG microscope (Houston, TX, USA).

UV-Vis-NIR transmission spectra of the BGB-xTb glasses (x = 8 and 9 mol% Tb_4_O_7_) were recorded in the spectral range 200–2000 nm using a Varian Cary 500 dual-beam spectrophotometer (Palo Alto, CA, USA).

The linear refractive indices of the BGB-xTb glasses were determined by the prism coupling technique using a Metricon 2010 M-lines system (Mount Waverley, Australia) at wavelengths of 532, 633, 972, 1308, and 1538 nm, with measurement precision of ±0.001.

The Faraday rotation angles (θ) of the BGB-xTb glasses (x = 8 and 9 mol% Tb_4_O_7_) and the core-cladding fiber were measured using a commercial NdFeB magnet (4.5 cm length and 1.5 cm diameter) under a 0.46 T magnetic field. The lengths (optical paths) of the bulk glass and core-cladding fiber samples were standardized at approximately 2 cm and 4.5 cm, respectively. The faces of the BGB-xTb (x = 8 and 9) samples were previously polished to obtain flat surfaces, while the core-cladding fiber was cleaved at the input and output edges using a cleaver machine (model FKII) (PK Technology, Beaverton, OR, USA). For fiber characterization, the sample was inserted into the Nd magnet and attached to a holder for positioning. A 40× objective lens was then used to focus a polarized laser beam on the input fiber section. The θ angles were measured at 650, 880, 1030, 1330, and 1550 nm using a SuperK Compact supercontinuum laser (Bengaluru, India) (spectral range: 450–2400 nm) with an output power of 100 mW at 15–30 °C and two graduated polarizers with an angular precision of ±2°. To ensure measurement precision, the rotation angles were determined five times for each sample. For measurements of the output beam at 650, 880, and 1050 nm, a handheld PM100D optical power meter (Thorlabs, Newton, NJ, USA) was employed. For the 1330 and 1550 nm wavelengths, detection was performed using a PDA015C InGaAs amplified photodetector (Thorlabs) connected to a handheld digital storage oscilloscope (Model 2512, 100 MHz, 1 GSa/s, BK Precision, Yorba Linda, CA, USA). The rotation angles (θ) were used to calculate the Verdet constant (V_B_) values (in rad T^−1^ m^−1^) using the Faraday equation (Equation (1)):

θ = V_B_. B. l (1)

where B (in tesla) is the magnetic field strength and l is the optical path length (in m).

Optical losses in the core-cladding fiber were determined by the cut-back method. For this, the fiber was sequentially cleaved to different lengths, ranging from an initial length of 75 cm to a final length of 25 cm, using a fiber cleaving machine. For each length, the output power was measured in the 350–1750 nm wavelength range using an optical spectrum analyzer (OSA) (Thorlabs, Newton, NJ, USA). The associated error was ±0.2 dB m^−1^.

## 3. Results and Discussion

### 3.1. Thermal, Viscosity, and Morphological Analyses

**Figure 2:**
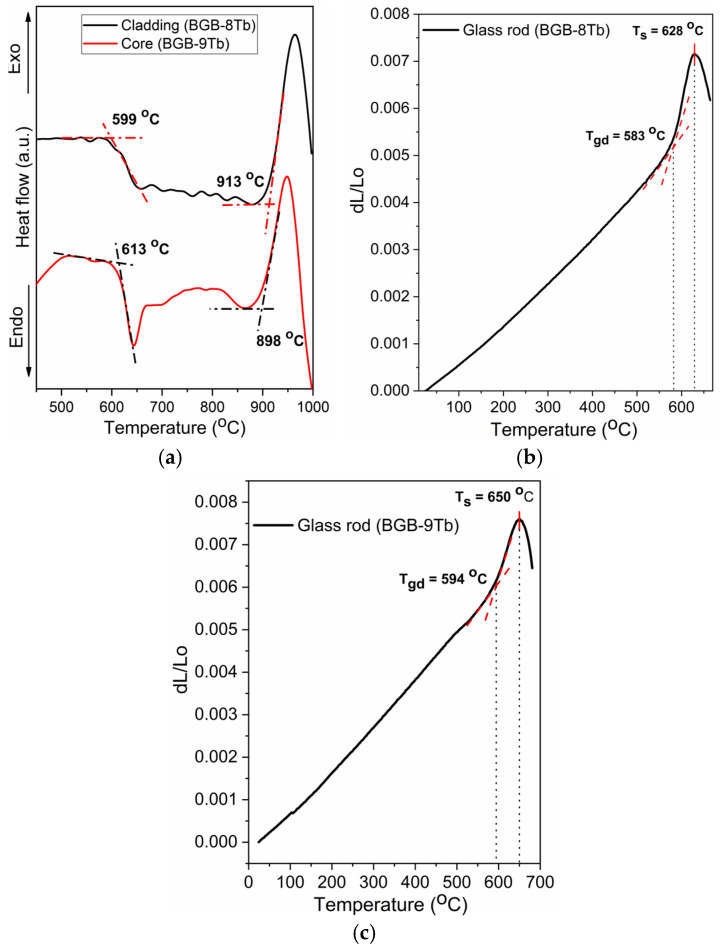
DSC curves of the BGB-xTb (x = 8 and 9 mol% Tb_4_O_7_) glass precursors (**a**) and TMA analyses of the (**b**) BGB-8Tb and (**c**) BGB-9Tb glass rods.

Figure 2a shows the DSC curves for the BGB-8Tb (core) and BGB-9Tb (cladding) glass precursors of the MO core-cladding fiber from which the glass transition temperatures (T_g_) were determined to be 599 and 614 °C, respectively. In addition, it is important to note that high ΔT values of 314 and 285 °C were obtained for the cladding (BGB-8Tb) and core (BGB-9Tb) compositions, respectively. A critical technological requirement in the production of high-quality optical fibers is adequate thermal stability of the glass precursors (ΔT > 100 °C) to avoid devitrification during the drawing process. Glasses containing high concentrations of RE ions often exhibit poor thermal stability and a tendency for crystallization. In this work, the high thermal stability of BGB glasses heavily doped with Tb^3+^ was the determining factor for selection of the compositions [22]. Table 1 summarizes the characteristic temperatures of the glass samples, including the glass transition (T_g_), crystallization onset (T_x_), dilatometric glass transition (T_gd_), and dilatometric softening (T_s_) temperatures, together with the thermal stability parameters (ΔT) and the coefficients of thermal expansion (CTE) in the 25–500 °C range.

The TMA results for the BGB-xTb (x = 8 and 9 mol%) glasses (Figure 2b,c) revealed that a higher Tb_4_O_7_ content led to higher T_g_ and T_s_ values. The CTE values of 10.3 × 10^−6^ K^−1^ (BGB-8Tb) and 10.2 × 10^−6^ K^−1^ (BGB-9Tb) in the 25–500 °C range were similar but suggested that an increase in the Tb_4_O_7_ content might act to reduce thermal expansion, reflecting enhanced structural rigidity of the glass network.

The increase in T_g_ according to Tb_4_O_7_ content was also indicative of enhanced structural rigidity of the BGB glass network, consistent with lower CTE (Table 1). Wang et al. found that the CTE values for soda-lime silicate glasses showed a dependence on the cationic field strength (CFS) of rare-earth elements (La, Ce, and Nd) [28]. Hence, the lower CTE observed here with an increase in the Tb^3+^ ion content could be explained by higher cationic field strength in the glass network. Franco et al. reported that higher density values of bulk BGB-xTb glasses were due to the high molar mass of Tb_4_O_7_ (747.70 g mol^−1^), while the glass transition temperature (T_g_) increased according to the Tb_4_O_7_ content [7]. Nemilov et al. [29] and Chen et al. [30] reported that increased rigidity of terbium-doped glass networks with increasing Tb_4_O_7_ content could be due to the higher bond energy of Tb-O (694 kJ mol^−1^) compared to Ge-O (352 kJ mol^−1^) and B-O (520 kJ mol^−1^). The T_g_ values obtained by both the DSC and TMA techniques showed that a higher Tb_4_O_7_ content led to increased rigidity of the glass network. The difference between the T_g_ values for the two techniques was due to the different heating rates employed (10 and 5 °C min^−1^ for the DSC and TMA methods, respectively).

**Figure 3:**
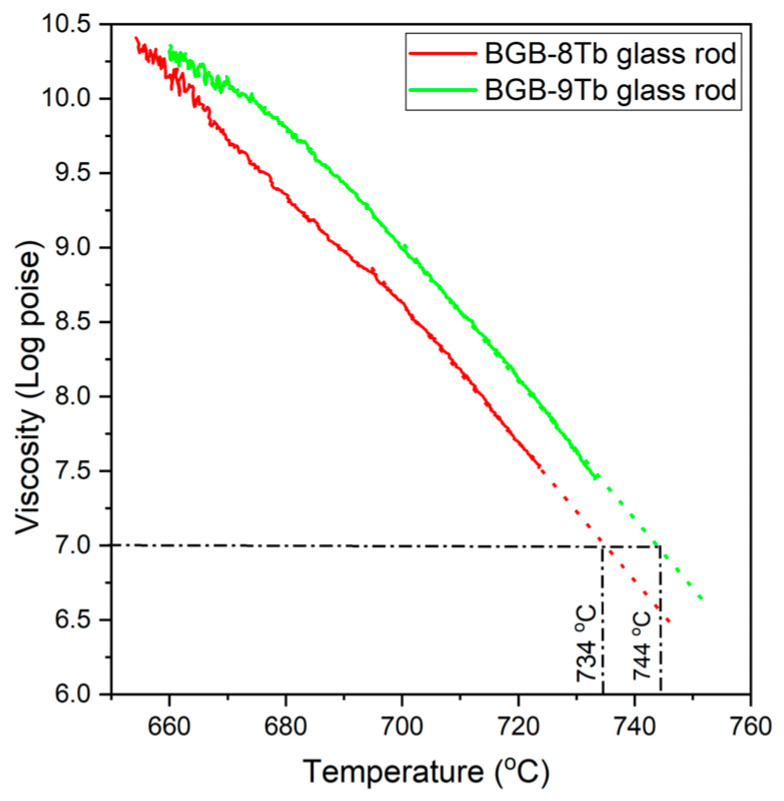
Viscosity-temperature curves for the BGB-xTb samples.

Figure 3 shows the viscosity-temperature curves for the BGB-8Tb and BGB-9Tb glass rods. The colored lines represent experimental data, while the black dotted lines indicate linear extrapolations [26,27]. Individual analysis of each glass composition was necessary to enable estimation of the temperature range suitable for fabrication of the core-cladding fiber. Additionally, linear extrapolation at 10^7^ poise enabled estimation of the fiber drawing temperatures (T_fib_), obtaining values of 734 °C for BGB-8Tb and 744 °C for BGB-9Tb (corresponding to viscosity of ~10^7^ poise). Interestingly, the experimental fiber drawing temperature for the BGB core-cladding fiber was approximately 740 °C.

The addition of Tb^3+^ ions to the BGB glass composition acted to increase the rigidity of the network, consequently leading to higher glass viscosity, as evidenced in the viscosity-temperature curve profiles (Figure 3) and from comparison of the T_fib_ values (Table 1).

**Figure 4:**
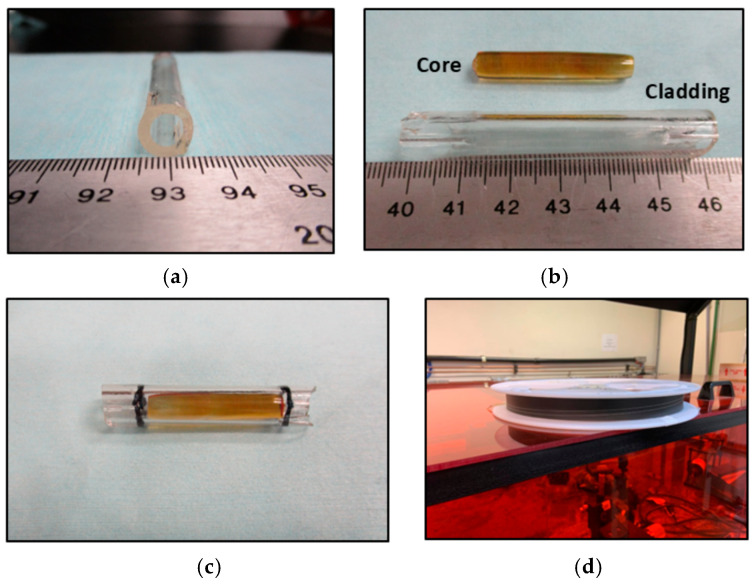
Photographs of (**a**) the cladding glass tube, (**b**) the core and cladding components, (**c**) the glass preform, and (**d**) the spool of core-cladding fiber obtained after drawing.

Figure 4 shows photographs of the cladding tube fabricated from BGB-8Tb glass, the core-cladding glass preform assembled with BGB-8Tb (cladding) and BGB-9Tb (core), and the spool containing the drawn core-cladding fiber.

The brownish coloration of the BGB-9Tb glass resulted from oxidation processes involving the conversion of Tb^3+^ to Tb^4+^, which becomes significant in glass compositions containing Tb_4_O_7_ at concentrations above 8 mol% [7,19,22,31,32].

**Figure 5:**
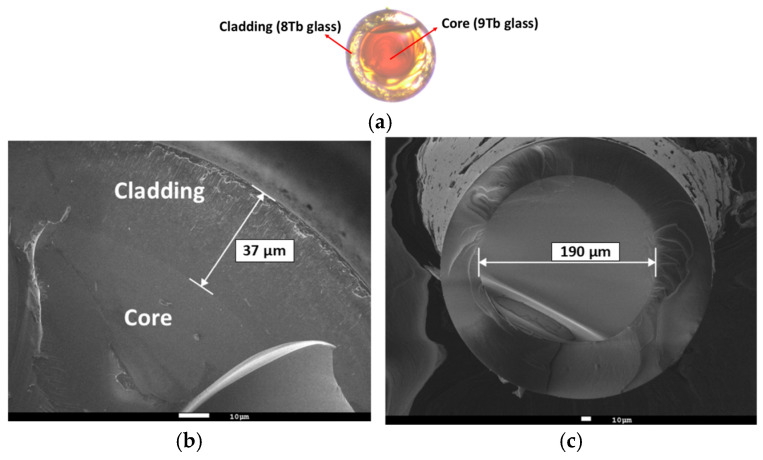
(**a**) Optical microscopy image and (**b**,**c**) scanning electron microscopy (SEM) images of the core-cladding fiber cross-section.

Figure 5a shows an optical microscope image at 50x magnification of the cross-section of the core-cladding fiber. Scanning electron microscopy (SEM) images of the core-cladding fiber cross-section are shown in Figure 5b,c.

The SEM images of the core-cladding interface morphology indicated that the cladding thickness was approximately 37 μm (Figure 5b), while the fiber core diameter was around 190 μm (Figure 5c).

### 3.2. Optical Analysis

**Figure 6:**
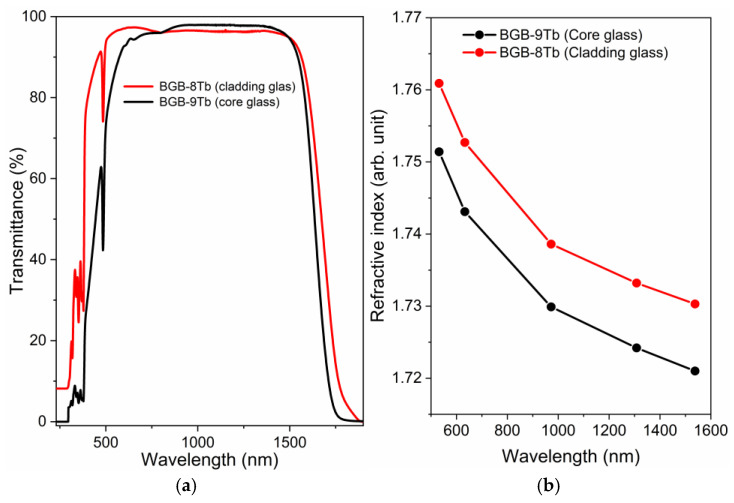
(**a**) Transmission spectra of the BGB-xTb glasses (x = 8 and 9 mol% of Tb_4_O_7_). (**b**) Refractive indices, as a function of wavelength, for the core (BGB-9Tb) and cladding (BGB-8Tb).

Figure 6a shows the transmission spectra of the core (BGB-9Tb) and cladding (BGB-8Tb) glass compositions. Absorption bands assigned to Tb^3+^ ions at 484, 377, 369, 351, 338, and 317 nm corresponded to electronic transitions from the ^7^F_6_ ground state to the energy levels ^5^D_4_, (^5^D_3_, ^5^G_6_), ^5^L_10_, (^5^G_4_, ^5^L_9_), (^5^G_2_, ^5^L_6_), and (^5^D_0,1_, ^5^H_7_), respectively [33]. Additionally, the precursor glasses exhibited an optical transmission window in the 500–1570 nm spectral range. The infrared transmission was limited by the Tb^3+^ transitions at around 1.85, 1.94, 2.1, and 2.46 μm, assigned to ^4^f–^4^f transitions from the ^7^F_6_ ground state to the ^7^F_0_, ^7^F_1_, ^7^F_2_, and ^7^F_3_ excited states, respectively [22].

Figure 6b shows plots of the refractive indices, according to wavelength, for the core (BGB-9Tb) and cladding (BGB-8Tb) compositions. The refractive indices were measured at 532, 633, 972.4, 1308, and 1537.7 nm. The values decreased with increasing wavelength, presenting normal dispersion behavior that became more pronounced at longer wavelengths. The values measured at 532 nm were 1.7609 ± 0.0001 for the BGB-9Tb core glass and 1.7514 ± 0.0001 for the BGB-8Tb cladding glass. The refractive index difference between the core and the cladding (Δn = n_core_ − n_cladding_) was 9.5 × 10^−3^. Theoretically, the confinement of light in the waveguide occurs by total internal reflection, which requires the core to have a higher refractive index than the cladding (n_core_ > n_cladding_) [34].

**Figure 7:**
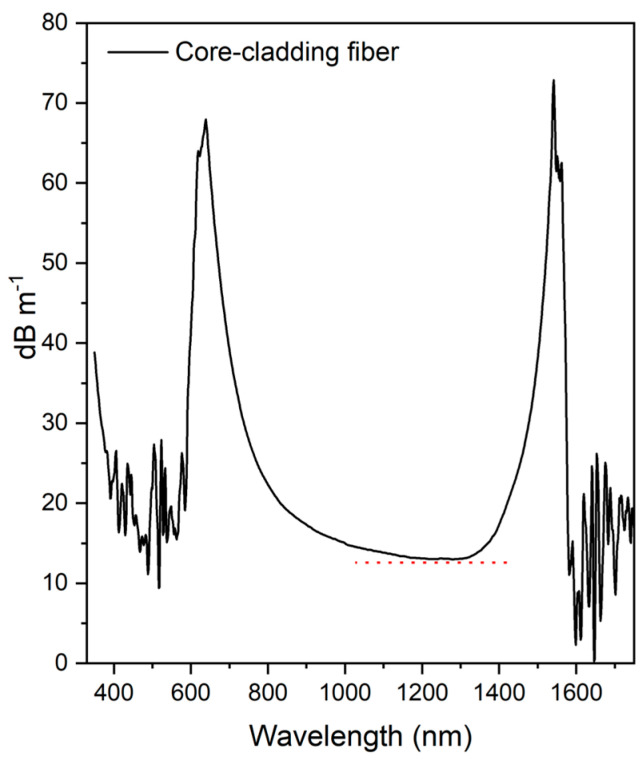
Attenuation spectrum of the core-cladding fiber obtained using the cut-back technique.

The attenuation spectrum of the core-cladding optical fiber (Figure 7) revealed two main optical loss regions at 350–500 and 1560–1700 nm, attributed to intrinsic Tb^3+^ ion absorptions, as shown in Figure 6a. Furthermore, it is important to highlight that there are absorption bands at around 630 nm and 1450 nm, attributed to water absorption, specifically to the third and second water overtones, respectively [35]. As discussed previously, borogermanate glasses exhibit intrinsic water absorption, which can compromise the optical properties of a fiber, particularly the transmission window in the visible to near-infrared (NIR) range. This absorption overlaps with the 1550 nm region, reducing the optical transmission window in the NIR. The presence of water in the fiber core can occur due to the intrinsic properties of the glass matrix, contamination during manufacture of the glass preform, or long-term environmental exposure. To minimize diffusion of water into optical fiber cores and the consequent signal loss, previous treatment of the raw materials must be performed to minimize water adsorption. This can be achieved by mixing the raw material powders with other chemicals at high temperatures (below the melting temperatures) or by more complex methodologies, such as distillation, as used for purifying chalcogenide precursors and subsequently to promote the melting of the glasses in a dry box, where the water content can be minimized by employing an atmosphere of N_2_ or Ar.

The minimum attenuation value of 13.0 dB m^−1^ at approximately 1270 nm could be attributed, as mentioned, to factors including the glass preform preparation method, impurities, strong water absorption, striae, and fiber imperfections, which can all contribute to loss of the optical signal.

The numerical aperture (*NA*) of the core-cladding fiber was determined using Equation (2) [36]:

(2) $$NA=\;\sqrt{n_{core}^{2}-n_{cladding}^{2}}$$

where *n_core_* and *n_clad_* are the refractive indices of the core (*n_core_* = 1.7609) and cladding (*n_clad_* = 1.7514), respectively. The calculated *NA* value was 0.183.

The critical angle (*θ_c_*) of the core-cladding fiber was determined using Equation (3) [36], obtaining a value of 84.04°:

(3) $$\theta_{c}={sin}^{-1}\frac{n_{cladding}}{n_{core}}$$

The acceptance angle (*θ_a_*) of an optical fiber, commonly referred to as the acceptance cone, defines the maximum angle at which incident light can propagate through the fiber core by total internal reflection. The *θ_a_* value was calculated according to Equation (4), using the refractive indices of the core (*n_core_*) and cladding (*n_cladding_*) [36], resulting in *θ_a_* = 10.5° for the fabricated core-cladding fiber:

(4) $$\theta_{a}=arcsin\left(\frac{1}{n_{0}}\sqrt{n_{core}^{2}-n_{cladding}^{2}}\right)$$

### 3.3. Magneto-Optical Measurements

**Figure 8:**
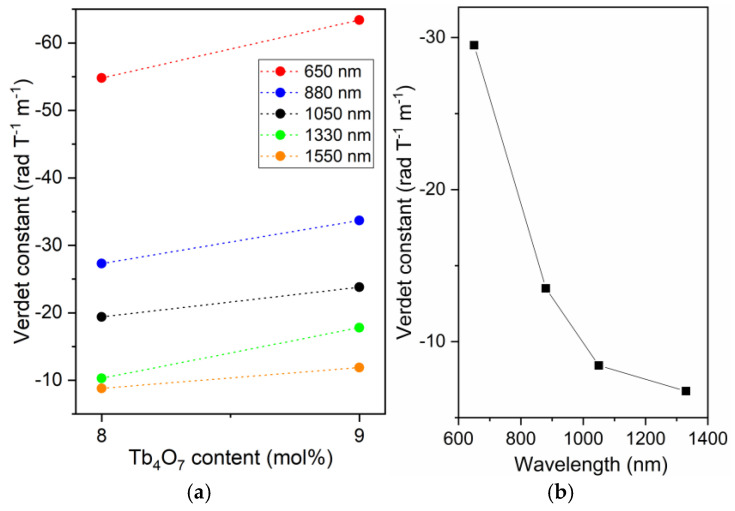
Verdet constant (V_B_) values as a function of (**a**) Tb_4_O_7_ content (mol%) for the core (BGB-9Tb) and cladding (BGB-8Tb) precursor glasses and (**b**) wavelength for the core-cladding fiber.

Figure 8a shows the variation in the Verdet constant (V_B_) according to the Tb_4_O_7_ content of the core (BGB-9Tb) and cladding (BGB-8Tb) precursor glass compositions, measured in the visible to NIR spectral range (500 to 1550 nm). Franco et al. reported V_B_ values in the 500–1550 nm wavelength range for the (100 − x)(41GeO_2_-25B_2_O_3_-4Al_2_O_3_-10Na_2_O-20BaO)-xTb_4_O_7_ glass system, where 0 ≤ x ≤ 18 mol% of Tb_4_O_7_ [22]. In this work, the V_B_ values for the BGB-8Tb and BGB-9Tb glasses at 650 and 1550 nm were −54.8 and −63.4 rad T^−1^ m^−1^ and −8.8 and −11.9 rad T^−1^ m^−1^, respectively. The small differences between the V_B_ values for the bulk glasses could be attributed to the higher Tb^3+^ concentration in the BGB-9Tb core glass composition, which resulted in higher V_B_ values at all wavelengths.

Figure 8b shows the experimental V_B_ values for the Tb^3+^-doped core-cladding fiber at different wavelengths. At 1330 nm, the V_B_ value was −6.74 rad T^−1^ m^−1^, corresponding to an approximately seven-fold enhancement, compared to a commercial SMF measured at 1310 nm (V_B_ = 0.876 rad T^−1^ m^−1^) [20]. Although total internal reflection occurred within the fiber core, the experimental V_B_ values differed from those of the precursor bulk glasses. For example, at 650 and 1330 nm, the V_B_ values for the core-cladding fiber and the core precursor glass were −29.5 and −6.74 rad T^−1^ m^−1^ and −63.4 and −17.8 rad T^−1^ m^−1^, respectively. This behavior was addressed in two earlier theoretical and experimental studies of the Faraday effect in single-mode fibers [24,37]. Yoshino et al. reported the first work demonstrating that the effect of modal field spreading in the core and cladding of a fiber can contribute to differences in V_B_ between the precursor glass and the core in single-mode optical fibers [37]. As a result, it was possible to calculate the so-called effective Verdet constant (V_eff_) theoretically, using experimental parameters such as the V_B_ values of the core and cladding and their respective refractive indices. In other words, this theory demonstrates that the total V_eff_ for the fiber differs from the values for the precursor bulk glass samples. Subsequently, Sun et al. reported theoretical calculations of V_eff_ using fiber parameters including experimental V_B_ values for the core and cladding bulk glass precursors of a 25 wt% terbium-doped core phosphate fiber with 6 wt% lanthanum-doped cladding [24]. In this case, the theoretically calculated V_eff_ for the Tb^3+^-doped core phosphate fiber was −6.2 ± 0.4 rad^−1^ T^−1^ m^−1^ (at 1053 nm), while V_B_ for the core precursor glass was −9.3 rad T^−1^ m^−1^. According to Barczak et al., few published works have described glass compositions with high thermal stability that are suitable for drawing into optical fibers for magneto-optical applications while also reporting their Verdet constant (V_B_) values [38]. In this work, although a multimode fiber was produced and characterized, the theoretical studies reported in the literature for single-mode fibers provided a useful basis for explaining the differences in V_B_ values between the bulk precursor glasses and the core-cladding fiber.

## 4. Conclusions

This work reports the design and fabrication of a core-cladding glass preform obtained by rotational casting and rod-in-tube techniques, followed by the successful drawing of a magneto-optical borogermanate fiber doped with a high concentration of Tb^3+^ ions. The thermal, optical, morphological, and magneto-optical properties of the precursor glasses and the terbium-doped core-cladding fiber were systematically investigated. The BGB-8Tb (cladding) and BGB-9Tb (core) compositions presented thermal stability parameters higher than 200 °C, showing that they were thermally stable against devitrification and making them excellent candidates for fiber manufacturing. After the drawing process, a magneto-optical core-cladding fiber with a 227 μm diameter was obtained. The fiber exhibited a minimum optical attenuation of 13 dB m^−1^ at 1270 nm and a numerical aperture of 0.183. In magneto-optical characterization, the core-cladding fiber presented a Verdet constant (V_B_) of −6.74 rad T^−1^ m^−1^ at 1330 nm. Nevertheless, there are significant possibilities for studying the magneto-optical applications of these fibers at 1310 nm, as this wavelength lies in the “O-band”, which is standard for short- to medium-distance communication links. In summary, this work successfully demonstrated the design, fabrication, and characterization of a Tb^3+^-doped borogermanate core-cladding optical fiber offering enhanced magneto-optical properties, with strong potential for use in advanced Faraday rotation devices.

## Data Availability

The original contributions obtained in this study are included in the article. Further enquiries can be directed to the corresponding author.

## Abbreviations

The following abbreviations are used in this manuscript:

RE	Rare-earth
SMF	Single-mode fiber
MMF	Multimode optical fiber
DSC	Differential scanning calorimetry
TMA	Thermomechanical analysis
UV-Vis-NIR	Ultraviolet-visible-near-infrared
NA	Numerical aperture
V_B_	Verdet constant
MO	Magneto-optical
TGG	Terbium gallium garnet
YIG	Yttrium iron garnet
NIR	Near-infrared
HMO	Heavy metal oxide
MCVD	Modified chemical vapor deposition
BGB	Borogermanate glass
PMMA	Poly(methyl methacrylate)
SEM	Scanning electron microscopy
OSA	Optical spectrum analyzer
CTE	Coefficient of thermal expansion
CFS	Cationic field strength
